# Mitigating Reptile Road Mortality: Fence Failures Compromise Ecopassage Effectiveness

**DOI:** 10.1371/journal.pone.0120537

**Published:** 2015-03-25

**Authors:** James H. Baxter-Gilbert, Julia L. Riley, David Lesbarrères, Jacqueline D. Litzgus

**Affiliations:** 1 Department of Biology, Laurentian University, Sudbury, Ontario, P3E 2C6, Canada; 2 Magnetawan First Nation, Britt, Ontario, P0G 1A0, Canada; Universidad de Granada, SPAIN

## Abstract

Roadways pose serious threats to animal populations. The installation of roadway mitigation measures is becoming increasingly common, yet studies that rigorously evaluate the effectiveness of these conservation tools remain rare. A highway expansion project in Ontario, Canada included exclusion fencing and ecopassages as mitigation measures designed to offset detrimental effects to one of the most imperial groups of vertebrates, reptiles. Taking a multispecies approach, we used a Before-After-Control-Impact study design to compare reptile abundance on the highway before and after mitigation at an Impact site and a Control site from 1 May to 31 August in 2012 and 2013. During this time, radio telemetry, wildlife cameras, and an automated PIT-tag reading system were used to monitor reptile movements and use of ecopassages. Additionally, a willingness to utilize experiment was conducted to quantify turtle behavioral responses to ecopassages. We found no difference in abundance of turtles on the road between the un-mitigated and mitigated highways, and an increase in the percentage of both snakes and turtles detected dead on the road post-mitigation, suggesting that the fencing was not effective. Although ecopassages were used by reptiles, the number of crossings through ecopassages was lower than road-surface crossings. Furthermore, turtle willingness to use ecopassages was lower than that reported in previous arena studies, suggesting that effectiveness of ecopassages may be compromised when alternative crossing options are available (e.g., through holes in exclusion structures). Our rigorous evaluation of reptile roadway mitigation demonstrated that when exclusion structures fail, the effectiveness of population connectivity structures is compromised. Our project emphasizes the need to design mitigation measures with the biology and behavior of the target species in mind, to implement mitigation designs in a rigorous fashion, and quantitatively evaluate road mitigation to ensure allow for adaptive management and optimization of these increasingly important conservation tools.

## Introduction

Increasing rates of urbanization, with associated habitat destruction and fragmentation, have led to the imperilment of much the world’s biodiversity [1, 2]. Roads and traffic present some of the longest lasting effects from both point-source mortality and enduring habitat and population fragmentation [1, 3]. The threats posed by roads extend from individual mortality to population-wide effects, as barriers within populations can lead to loss of genetic diversity and isolation [4]. Over the last two decades, the field of road ecology has grown to include examination of numerous taxa and incorporates a wide variety of disciplines, all with the common goal to better understand the interaction between roads and wildlife [1, 5, 6, 7]. Closely tied to this research effort is the development of mitigation strategies aimed at protecting wildlife from the negative effects of roads [8], with particular focus on large-bodied species [9, 10]. Far fewer studies have examined effectiveness of road mitigation for small-bodied species [11, 12]. Reptiles, considered one of the most imperilled groups of animals globally [13], have been substantially affected by the proliferation of roads [14, 15, 16], and conservation strategies to minimize threats posed by roads are being implemented [17, 18]. Yet, few studies have evaluated the effectiveness of these conservation efforts [7].

Threats to reptiles from roads are multifaceted and often relate to the species’ specific ecological and life-history traits, behaviors, and movement patterns [19, 20, 21]. Turtles regularly encounter roads during long-distance seasonal movements, and road mortality of adults leads to population declines because of the “bet-hedging” life history of turtles that requires high adult survivorship for population persistence [14, 22]. Turtles are particularly susceptible because up to 98–100% of individuals can be killed during their first road-surface crossing attempt [18]. Similar to turtles, seasonal movements of snakes also require road crossings, and road mortality has been identified as a population-level threat to several species [23, 24, 25]. Snakes bask on road surfaces to absorb radiant heat; this behavior prolongs exposure to traffic and increases the likelihood of collisions [26]. Further increasing the threat to reptiles, 2.7% of drivers will intentionally run over snakes [27].

Mitigation measures have been designed to reduce road mortality by installing exclusion structures (e.g., fences, gravity walls), and to reduce fragmentation by installing population connectivity structures (e.g., ecopassages, bridges) [17, 18, 27]. The integration of such structures into highway designs is becoming increasingly common for a wide variety of affected wildlife, yet the effectiveness of these mitigation measures is rarely quantified [7, 19, 28, 29]. This lack of assessment is a concern because functional and cost-effective conservation measures are critical to the recovery of imperiled populations, especially given the limited funds available for conservation projects [7]. Evaluation is needed to provide a framework for effective and logistically feasible mitigation that can be regularly implemented into roadways [6, 30, 31].

Effective roadway mitigation measures must meet the following criteria [19, 28, 32]: 1) reduction in abundance of wildlife on roads, 2) maintenance of habitat connectivity and dispersal routes, and 3) prevention of prey-trap formation. To this end, we quantitatively assessed these mitigation criteria for an assemblage of reptiles along a major roadway in Ontario, Canada. Our study used these criteria to rigorously assess the effectiveness of the mitigation via four methods: i) Before-After-Control-Impact (BACI) study to examine change in reptile abundance on roads, ii) radio telemetry to examine reptile movements around roads, iii) a willingness to utilize (WTU) experiment to assess likelihood of ecopassage use, and iv) monitoring ecopassages using wildlife cameras and an automated passive integrated transponder (PIT-tag) reading system to quantify reptile and predator presence. If the exclusion structures are effective at preventing reptiles from accessing the highway, we expect a significant decrease in the abundance of reptiles on the highway post-mitigation (criterion 1; method i). Concurrently, if the connectivity structures are effective at promoting population and habitat connectivity, then we expect that individuals should use the ecopassages to gain access to resources on either side of the highway without exposing them to an increased risk of mortality from collisions with vehicles or potential predators within ecopassages (criteria 2–3; methods ii-iv).

## Methods

### Study area and mitigation measures

Mitigation measures were constructed to reduce reptile road mortality and provide safe crossing options along a newly expanded section of Highway 69/400 in central Ontario, Canada. This major thoroughfare runs north-south and bisects the Georgian Bay coastline of Lake Huron, one of Canada’s richest areas of reptile biodiversity [33]. In addition, the area has a high number of reptiles designated as species at risk (SAR [34]). The highway expansion and associated increase in traffic present long-lasting and significant threats to 6 species of turtles (5 SAR) and 12 species of snakes (5 SAR) in the region [34].

Our study was conducted over two years at two sites located 50 km apart: (1) an Impact site near Burwash, Ontario, Canada and (2) a Control site at Magnetawan First Nation. The survey areas at both sites consisted of a 13-km section of Highway 69/400 with comparable reptile diversity [34], habitats (i.e., wetland mosaics interspersed with upland rocky outcrops and mixed forests), and traffic volumes [35]. The Impact site was a 2-lane un-mitigated highway during 2012 and a 4-lane mitigated highway in 2013. The Control site, a 2-lane highway, remained un-mitigated during both study years.

**Fig 1:**
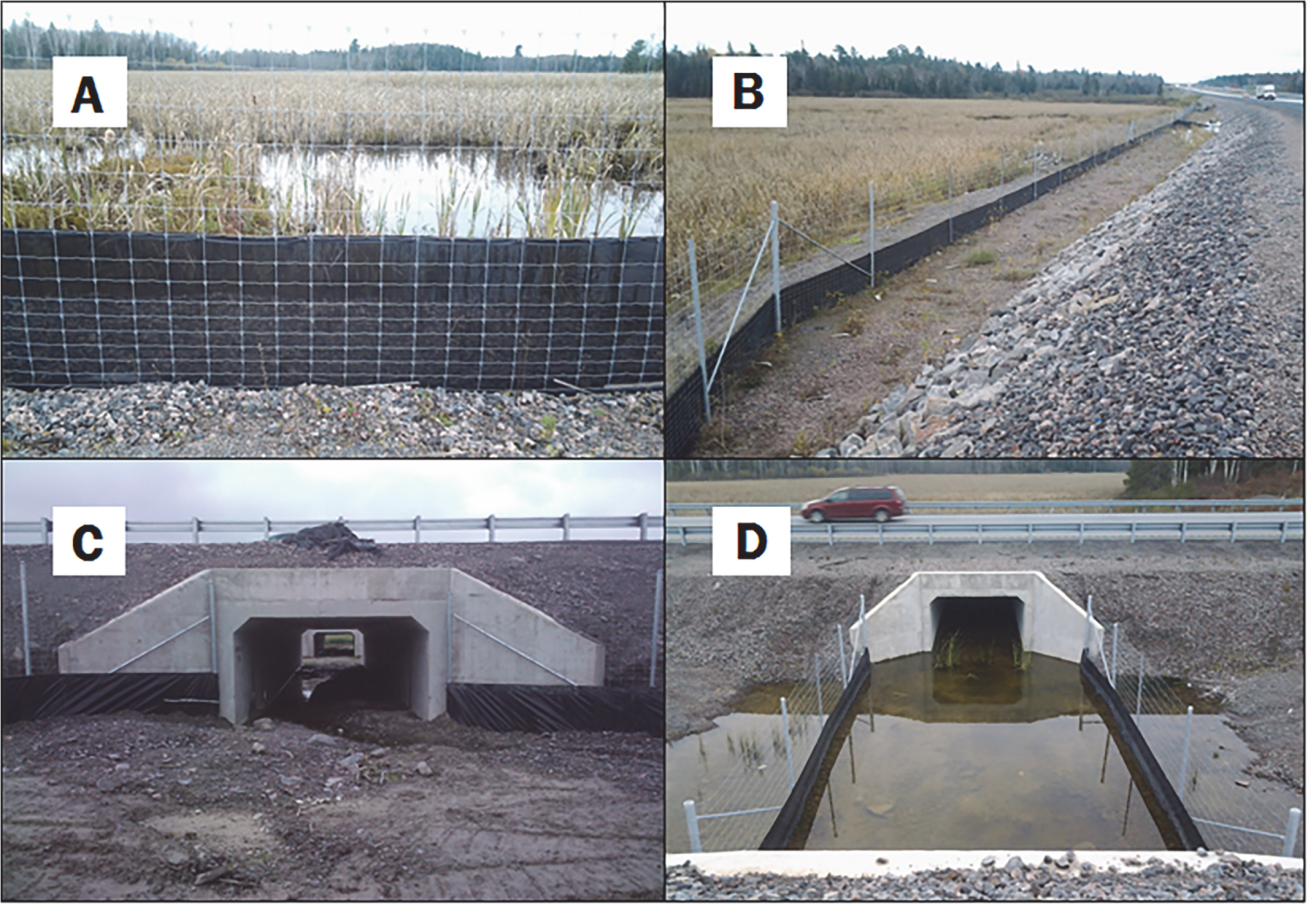
Mitigation measures completed during the fall of 2012 along Highway 69/400 in central Ontario, Canada. These measures include reptile fencing consisting of a heavy gauge plastic geotextile extending 0.8 m above- and 0.2 m below-ground with a 0.1 m wide lip running perpendicular underground (A). The fence was affixed to a 2.3 m tall large mammal, wire fence and was installed in areas believed to pose a risk to reptiles (B). Three ecopassages were built within the fenced area and each consists of two 3.4 m x 2.4 m x 24.1 m concrete box culverts (C), separated by a 15.3 m gap for increased light (D).

The mitigation measures at the Impact site consisted of an exclusion structure (reptile fencing) and three population connectivity structures (ecopassages). The reptile fencing consisted of heavy-gauge plastic textile extending 0.8 m above- and 0.2 m below-ground with a 0.1 m wide lip running perpendicular underground. The fence was affixed to the base of a 2.3 m tall chain-link fence intended to exclude large mammals from the highway (Fig. 1A and 1B). Sections of reptile fencing were installed along the highway at the Impact site in areas that were identified as important habitat and to be potential hotspots for reptile road crossings. The reptile fence connected the three ecopassages (spaced 450–600 m apart), and extended beyond the north ecopassage by 600 m and beyond the south ecopassage by 150 m. Each ecopassage consists of two 3.4 m x 2.4 m x 24.1 m concrete box culverts that cross the north-, and south-bound lanes of the highway (Fig. 1C). A fenced 15.3 m gap connects each culvert through the median between the lanes (Fig. 1C), allowing ample light to enter the ecopassages [36].

All summary data are reported as means, followed by 1 SE. The significance level of α = 0.05 was used for all statistical tests, and all statistical analyses were conducted in R (version 2.15.0, R Development Core Team, 2012).

### Effectiveness of the exclusion structures

#### BACI study (criterion 1)

The BACI study examined the differences in abundance of reptiles on the highway between the Before (2012) and After (2013) periods. Simultaneous surveys were conducted by car at the Impact and Control sites three times daily (09:00, 18:00, 22:00) from 1 May to 31 August each year. Daily roadside walking transects (RWT; at approximately 10:00) were conducted on foot and covered 2 km of highway. At the Impact site, the RWT was located in an area with continuous mitigation (in the After period, 2013), while the RWT at the Control site spanned an area with habitats similar to those at the Impact site. Over the two-year study period, a total of 1,934 surveys were conducted. Location and mortality status were recorded for any reptile found on the highway during both the driving and RWT surveys. To determine if individuals were being recaptured, each live reptile captured on the highway was individually marked and released at a safe distance off the highway in the direction it was heading. If the reptile was deceased, the individual was removed from the road to avoid being re-counted.

Differences between reptile abundances on the highway between the Before and After periods were examined using a Poisson generalized linear model (GLM) for non-parametric count data, including the fixed effects of period (Before, After), site (Control, Impact), and the interaction between these two effects. To ensure consistency in findings, differences in relative abundances of snakes and turtles at the two sites were also compared between Before and After periods by a paired two-sample t-test. The percentage of dead reptiles detected out of the total reptiles recorded on the road during surveys was also calculated at the Control and Impact sites during both the Before and After periods.

### Effectiveness of the connectivity structures

#### Radio telemetry (criterion 2)

The spatial ecologies of two SAR turtles at the Impact site were studied using radio telemetry to examine animal movements, road crossings, and to determine average dispersal distance (the linear distance an individual would travel between crossing structures or to circumvent exclusion structures during a movement event, calculated as the square root of home range size [37]). During both the 2012 and 2013 active seasons, adult Blanding’s turtles (*Emydoidea blandingii*, *n* = 10, threatened [34]) and snapping turtles (*Chelydra serpentina*, *n* = 12, special concern [34]) captured within 1 km of the highway were outfitted with radio transmitters (R1920, Advanced Telemetry Systems). Individuals were tracked every 2 to 3 days and locations were recorded using a handheld GPS unit (eTrex Vista, Garmin).

Home range sizes (95% minimum convex polygons [38]), and the number of highway crossings per individual were calculated and tallied (ArcGIS 10.0, ESRI). Home range sizes did not differ between species (*F*_1,27_ = 0.15, *p* = 0.70), sexes (*F*_1,27_ = 0. 01, *p* = 0.93), or the Before and After periods (*F*_1,27_ = 0.01, *p* = 0.92) allowing the data to be pooled to create an average home range size for all turtles. Average home range size was then used to calculate a local SAR turtle-specific dispersal distance [37]. Individual home ranges overlapping with the highway post-mitigation and road-crossing locations were counted for all radio-tracked turtles.

#### Willingness to utilize experiment (criterion 2)

Between 1 May to 31 August 2013, adult painted turtles (*Chrysemys picta*; males *n* = 24; females *n* = 30) were collected from a wetland 2.5 km west of the highway at the Impact site. Individuals were transported to a testing site at the east entrance of an ecopassage. Painted turtles were used for the willingness to utilize (WTU) experiment because of their ability to navigate using the sun [39, 40], and their ability to return to their home range [41]. During the experiment, the ecopassage was located between the individual (east of the highway) and its home wetland (west of the highway), creating a scenario in which a turtle was motivated to move in a specific direction similar to seasonal movements to critical habitat (e.g., overwintering or nesting sites). Also, painted turtles were used for behavioral trails because we could capture them in high numbers, they are frequently found on roads [42], and because previous studies have examined their willingness to use ecopassages in laboratory and arena settings [36, 43].

Prior to the experiment, individuals were temporarily outfitted with a radio transmitter (R1680, Advanced Telemetry Systems) and placed in an acclimation box 5 m from the entrance of the ecopassage. The turtle was left to acclimate to the sun’s position (to provide a cue for navigation), the substrate, and the noise and smell of the highway for 10 min. After the acclimation period, the box was remotely opened by a researcher situated behind a blind [20]. Turtle movements were monitored from behind the blind to assess the individual’s interactions with the ecopassage. Each individual’s behavior was ranked using a measure of crossing success on a scale from 0–2: 0) not willing to use, walked away; 1) made no choice, remained at entrance; 2) willing to use, crossed into, or through, the culvert [44]. After 20 min, or if an individual moved greater than 10 m out of the testing area, the turtle was collected and its location was recorded. All WTU tests occurred within less than 8 h of capture. After the WTU test, the turtle was marked to prevent reuse, and returned to the original site of capture within 12 hours.

Due to the importance of connectivity within populations for both sexes [45], and because no difference was detected between the sexes in WTU scores (*χ* ^2^_2_ = 0.79, *p* = 0.67), the data were pooled for analyses. A Pearson chi-squared test was used to compare WTU scores from our experiments to scores for turtles tested in an arena study (*n* = 190 [43]). The proportion of turtles in our study that refused to use the ecopassage (scored 0), that made no decision regarding the ecopassage (scored 1), and that were willing to use the ecopassage (scored 2) were compared to similar data obtained during the first 30 min of testing conducted in the arena study (91/190 (48%) refused, 10/190 (5%) no decision, 89/190 (47%) used ecopassages [43]).

#### Ecopassage monitoring (criteria 2 & 3)

Wildlife cameras (TrophyMAX, Bushnell) were installed in the entrances of the ecopassages to monitor use by reptiles and potential reptile predators [17, 11]. Cameras were mounted to the ceiling of the culvert, and aimed directly at the ground to maximize frame coverage. During the day, the motion-sensors set at the highest sensitivity, triggered photo capture. Because slow-moving terrestrial ecotherms are difficult to detect with infrared motion sensors at night, cameras were programmed to automatically take a picture every minute between the hours of 1830 to 0630 [11].

Additionally, automated passive integrated transponders (PIT-tag) readers (HPR, Biomark) with loop antennas (BIO 10 Antenna, Biomark) were installed in the central ecopassage at the Impact site prior to the 2013 field season. The antennas spanned both entrances of the ecopassage, providing information on the number of individuals entering the ecopassage, the number successfully exiting the other side, and duration of the crossing event. The readers constantly scanned and logged the PIT-tag number of any animal that passed through the loop antenna, and the date and time of crossing. During the 2013 active season, adult turtles (*n* = 38: 6 painted turtles; 15 Blanding’s turtles; 17 snapping turtles) and snakes (*n* = 20: 8 eastern gartersnakes, *Thamnophis sirtalis;* 12 northern watersnakes, *Nerodia sipedon*) found within 1 km of the highway were captured and individually marked with a subcutaneously-injected PIT-tag (HPT12, Biomark).

All research was conducted under approved Laurentian University Animal Care Committee protocols (AUPs 2008–12–02 and 2013–03–01, and was authorized by Magnetawan First Nation’s Chief and Council and the Ontario Ministry of Natural Resources.

## Results

### Effectiveness of the exclusion structures

#### BACI study (criterion 1)

A total of 960 road surveys were conducted in 2012, and 974 were conducted in 2013. We recorded 618 snakes and 378 turtles on the highway at both sites combined, and the percentage of dead snakes detected on the road was 83% and for turtles was 84%. In all cases, 2–9 times more animals were found dead on the road than alive, depending on site and time period (Table 1). These levels of mortality are far higher than would be considered sustainable for many reptile species—especially snapping turtles based on their life history [46] and known population densities at this latitude [47]. Alarmingly, the percentage of dead reptiles detected at the Impact site increased by 20% for turtles and 25% for snakes between the Before and After periods, while the Control site had an increase of only 2% for turtles and 11% for snakes.

**Table 1:** Number (proportion) of reptiles, alive (AOR) and dead (DOR), observed on the road between samples periods (Before, After) and sites (Control, Impact).

Taxa	Before				After			
	Control		Impact		Control		Impact	
	AOR	DOR	AOR	DOR	AOR	DOR	AOR	DOR
Turtle	20 (14%)	121 (86%)	18 (32%)	39 (68%)	15 (12%)	108 (88%)	8 (14%)	49 (86%)
Snake	41 (24%)	131 (76%)	26 (32%)	55 (68%)	34 (12%)	261 (88%)	7 (10%)	63 (90%)
Total	61	152	44	94	49	369	15	112

**Fig 2:**
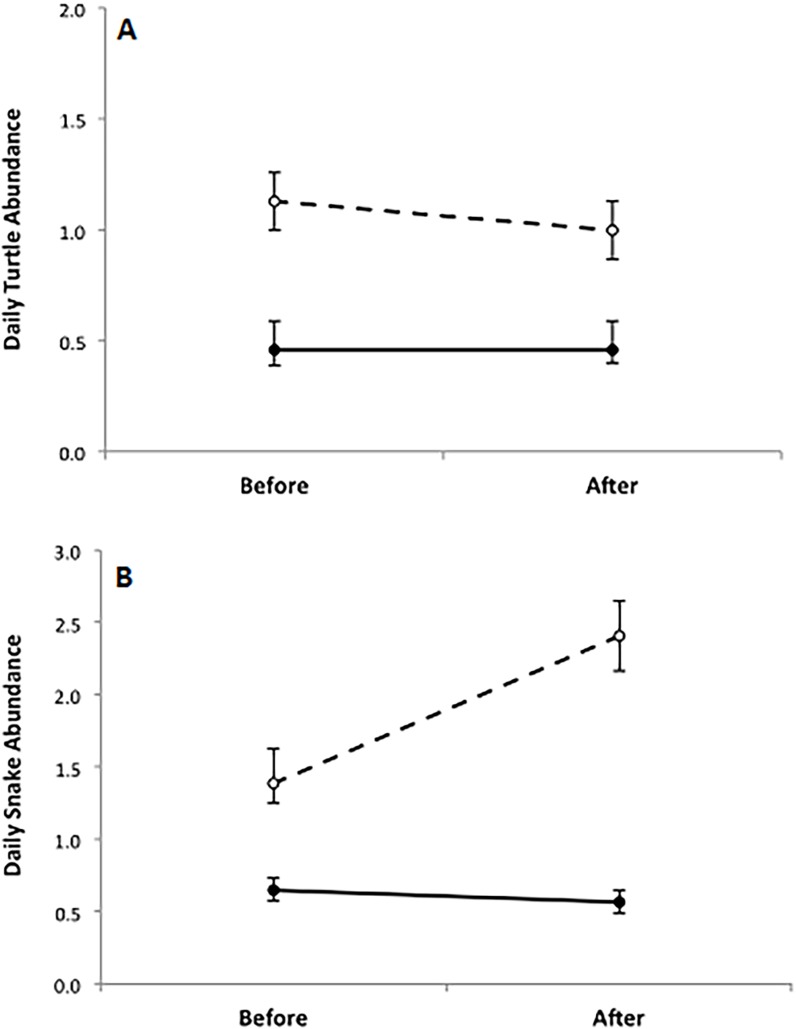
Daily abundance of reptiles on the highway for each survey period (Before and After) did not differ for turtles (A), but did differ for snakes (B) when considering survey sites (Impact (●) and Control (○)). The parallelism between the solid and dashed lines visually represents no significant interaction between site and period for turtles (A; GLM *z*_488 =_ -0.05, *p* = 0.57), while this interaction was significant for snakes (B; GML *z*_488 =_ 3.60, *p* < 0.01). Yet, a strong reduction in snake abundance at the Impact site was still not seen between periods, thus the interaction is due to the large increase in snake abundance observed at the Control site during the After period.

The Poisson GLM demonstrated no significant interaction between period (Before and After) and site (Control and Impact) for turtles (*z*_488 =_ -0.05, *p* = 0.57) but there was a significant interaction for snakes (*z*_488 =_ 3.60, *p* < 0.01); however, this interaction was due to an increase in snakes recorded at the Control site rather than a decrease in snake presence at the Impact site. The paired two-sample t-test corroborated our findings. Relative turtle abundance (Control relative to Impact) on the highway did not differ between the Before and After periods (*t*_121_ = 0.81, *p* = 0.42) (Fig. 2A). In contrast, relative snake abundance on the highway differed between the Before and After periods (*t*_121_ = -3.78, *p* < 0.01); however, snake abundance on the road was not substantially reduced at the Impact site post-mitigation and the statistical difference between the Before and After periods is attributed to the increase in snake abundance on the road at the Control site (Fig. 2B).

### Effectiveness of the connectivity structures

#### Radio telemetry (criterion 2)

The average home range size for all turtles combined was 42.5 ha (SE 12.4), and the calculated dispersal distance was 652 m. Post-mitigation, there were 11 road crossings by 3 of the turtles (2 snapping turtles, and 1 Blanding’s turtle) and 1 snapping turtle passed through the exclusion fence but did not cross the highway. Based on telemetry locations, the successful road crossings were not likely through an ecopassage, but rather through a drainage culvert that was incorporated into the mitigation fence.

#### Willingness to utilize (WTU) test (criterion 2)

Most turtles did not make a decision regarding use of the ecopassages within the allotted time (*n* = 37/54; 69%). Of the individuals that did make a decision, more than twice as many turtles refused to use the ecopassage (*n* = 12/54; 22%) than were willing to enter (*n* = 5/54; 9%). When our results were compared to those from a previously reported arena study [43], we found that far fewer individuals were willing to use an ecopassage below an active highway than in a testing arena (*χ* ^2^_2_ = 863.52, *p* < 0.001).

#### Ecopassage monitoring (criteria 2 & 3)

A total of 485 individual animals were photographed in the ecopassages, consisting of at least 23 non-reptile and 3 reptile species (S1 Table). Ducks and geese (Family Anatidae) were present in 40.2% of photographs and were the most common taxa recorded using the ecopassages. In contrast, reptiles were one of the least photographed taxa (in 2.0% of photos). Painted turtles were photographed in the ecopassages on 6 occasions (4 adults and 2 hatchlings; 1.2% of photos). An adult snapping turtle (0.2% of photos), and 3 northern watersnakes were also photographed in the ecopassages (0.6% of photos). Additionally, during regular camera maintenance, snapping turtle tracks were observed that were not associated with a photograph, and a live juvenile red-bellied snake (*Storeria occipitomaculata*) was also observed within an ecopassage. A number of potential reptile predators were also seen using the ecopassages: *Ardea herodias* (8.9% of photos), *Procyon lotor* (8.2%), *Neovison vison* (2.3%) and *Canis latrans* (1.7%).

Although frequent tests of the automated PIT-tag reader occurred, only two PIT-tagged animals were recorded in the ecopassage: a watersnake and a painted turtle. In both cases, the readers did not detect a complete crossing (i.e., PIT-tags were not logged at both entrances). Instead, it appears that the individuals either retreated from the entrance of the ecopassage after approaching the reader, or circumvented the exclusion fencing within the highway median between the ecopassages.

## Discussion

To determine the effectiveness of exclusion and connectivity structures, we examined whether the mitigation resulted in a reduction in abundance of reptiles on roads (criterion 1), while maintaining habitat connectivity and dispersal routes (criterion 2) without forming a prey-trap (criterion 3). By taking a broad, multispecies approach we were able to examine effectiveness of the mitigation to determine if it had the desired wide-ranging ecological impact (i.e., across multiple species and demographic groups). We found that the success of the connectivity structures was reliant upon the success of the exclusion structures, and that the entire mitigation system was compromised at our site because of failures in the materials, implementation, and design of the exclusion structures. Our findings demonstrate the importance of designing robust, biologically-relevant exclusion structures to mitigate road mortality of a variety of small-bodied terrestrial and semi-aquatic animal taxa.

### Lack of reduction in abundance of reptiles on the road (criterion 1)

The first criterion of our evaluation was not satisfied because the reptile fence did not prevent turtles from gaining access to the road surface, and the percentage of dead turtles detected on the road increased by 20% during the post-mitigation period. In contrast, surveys post-mitigation did record a difference in relative abundance of snakes on the road; however, this difference was not due to a decrease in the abundance of snakes on the highway at the Impact site, but rather due to the prevention of a parallel proportional increase in snake abundance as observed at the Control site in 2013. Furthermore, there was a 25% increase in percentage of dead snakes detected on the road at the Impact site in the post-mitigation period, indicating that although the fence may have prevented a proportional increase in abundance on the road, as seen at the Control site, many more individuals were killed on the highway post-mitigation.

The increase in the percentage of dead turtles and snakes detected on the road post-mitigation may be attributed to a corralling effect of the exclusion fence on individuals who gained access to the highway via fence-gaps. Reptiles may then be forced to spend an increased amount of time on, or adjacent, to the highway in search of a gap in the fence through which to pass on the other side of the road [48]. Another potential cause of the increase in the percentage of dead reptiles on the road may have been the increase in road surface area from 2-lanes pre-mitigation to 4-lanes post-mitigation. Although increases in road width may increase reptile exposure to traffic, one of the goals of mitigation is to reduce road mortality and if an overall reduction is not seen, then mitigation cannot be deemed effective regardless of the changes in road width. In fact, in many cases, highway improvement projects involve the widening of highways, thus our results are representative of what wildlife is truly experiencing on the landscape. Considering that there was no relative reduction in turtle abundance, and an increase in both snake and turtle mortality were observed, we conclude that a non-continuous, flexible-plastic fence is not capable of reducing reptile abundance on a highway. The inability of the fencing tested in our study should not be generalized to all exclusion structures; several studies have documented high success rates with various styles of exclusion structures [17, 18]. This begs the question, why was the fence in our study not effective?

Close examination of the fence revealed a suite of issues that rendered this style of exclusion structure incapable of preventing reptiles from gaining access to the highway, including failures in both the material and installation. One of the main issues was over 115 gaps located along the 3 km of sectionally-fenced highway resulting from rips, holes, and washouts. Furthermore, during the spring melt, up to 30% of the fence was semi-submerged, which allowed reptiles to easily swim or climb over the fence. Between areas that were deliberately left unfenced and the many unintentional gaps, approximately 2/3 of the sectionally-fenced area was permeable. The distance between locations of reptiles found on the highway in the fenced area (*n* = 91) to the closest known gap in the fence averaged only 38.3 m (SE 4.2). This is a relatively small distance for reptiles to cover given their spatial ecologies [25, 49], clearly indicating that animals were easily gaining access to the road through the gaps.

The need to increase the effectiveness of exclusion structures is evident, particularly in light of both the high levels of road mortality documented and the permeability of the type of fence used in our study. We suggest the use of more durable materials in the design of exclusion structures. Flexible-plastic fencing is prone to rips and tears, quickly degrades over the short-term, and requires regular maintenance [18]. Also, both plastic and metal mesh fences are easily climbed by many reptile species [18, 50]. High water levels and drainage must be taken into consideration so that the threats of washouts and flooding are minimized, as exposure to water will degrade or destroy exclusion structures. Roads are built to be long-lasting structures, and mitigation measures should be equally long-lasting. An alternative exclusion structure to fencing would be a concrete or steel gravity wall fitted into the sloped gravel between the shoulder and ditch, which would provide a solid long-lasting barrier known to be effective [17]. Although incurring a higher initial cost, this more durable exclusion structure may be far more cost-effective compared to intense maintenance (e.g., annual maintenance costs can equal installation cost of geotextile fencing [51]) that is necessary to maintain this type of fencing over the long term [18]. Furthermore, as previous evaluation indicates [17], concrete or steel gravity walls are more biologically effective (i.e., they successfully reduce road mortality of animals), which is a crucial consideration in order to effectively protect imperiled, ecologically-important species. When planning mitigation measures it is also important to take a pragmatic approach, such as using a cost-benefit analysis [52] including consideration of factors such as: initial cost, maintenance costs, human-wildlife collision costs, effectiveness, benefits to target species, overall ecological benefit, human benefit, and the lifespan of the structure. The relative weighing of the factors used to determine what style of mitigation is warranted and effective for ungulates can be distinctly different than those for reptiles, particularly because mitigation measures for ungulates are often implemented to off-set the costs of human-wildlife collisions [52], rather than as a conservation tool. When the goal of mitigation measures is conservation, then long-lasting exclusion structures are required to ensure that conservation efforts are successful at reducing the abundance of wildlife on roadways to a level that is biologically significant for population viability.

### Maintenance of habitat connectivity and dispersal routes (criterion 2)

The ecopassages demonstrated low use, and likely would have had more crossings if the exclusion fencing was functioning properly. The wildlife cameras and haphazard encounters showed that 4 reptile species used ecopassages. However, the number of observations documented within the ecopassages (*n* = 12) was much lower than the number of individuals found on the highway during the same timeframe (*n* = 127). Additionally, data from the two automated PIT-tag readers detected only 2 of the 54 individual reptiles implanted with PIT-tags [42]. This further indicates that although reptiles were observed using the ecopassages to facilitate movements between or within habitats, the number of reptiles using the ecopassage to cross was only 9% of the number using the road surface to cross.

Our two-year radio telemetry study of Blanding’s and snapping turtles at the Impact site established that home range sizes did not change in response to the changes in the highway and that 27% of the radio-tagged individuals had home ranges that overlapped with the highway (*n* = 6/22). These individuals had critical habitats (i.e., nesting and overwintering sites) and seasonal habitats (i.e., basking and foraging sites) on both sides of the highway. The locations of road crossings, used to move between critical and seasonal habitats, were all within the calculated dispersal distance of the turtles in our study (652 m) to an ecopassage, suggesting that although the turtle had access to ecopassages, they choose not to use them. In fact, alternative methods of crossing were used that involved circumventing the fence (*n* = 1/4 turtles), or crossing via a drainage culvert (1.0 m diameter; *n* = 3/4 turtles) incorporated into the fence. The use of the drainage culvert is believed to be a result of its placement in an open channel in the center of the wetland, an observed movement corridor, rather than a preference by individuals towards small, flooded, poorly-lit drainage culverts. The use of the drainage culvert rather than the large well-lit ecopassages illustrates the importance of carefully selecting ecopassage locations. If ecopassages are constructed for only a limited number of species, then specific habitats can be targeted; however, if the goal is to mitigate for a wide variety of species, or if habitat selection by a target species varies due to sex and age-class, then diversifying ecopassage locations (e.g., movement corridors in wet and dry, lowland and upland locations) may assist in maximizing likelihood of use. Overall, the turtles did not use the ecopassages as travel routes, which together with the low use by reptiles in general, translates to a failure in this criterion of effectiveness.

The WTU test provided valuable insight regarding the lack of ecopassage use observed during our study as turtles were twice as likely to refuse using an ecopassage as they were to use it. This was significantly different from results obtained in an arena study [43] and we suggest that such differences may be because we tested ecopassage use beneath a live highway with the sights, sounds, and smells of traffic. Our findings thus provide a more realistic understanding of a turtle’s response to an ecopassage. Although the previously reported arena study also tested variable culvert apertures [43], all of which were smaller than that in our study, the fact that turtles were less likely to use a large, well lit ecopassage with ample natural substrate was a surprising result. The relatively higher usage observed in arena studies may have been driven by the experimental design in which the turtle was only provided a single option—moving through the ecopassage (simulating a crossing) with no opportunity to escape [36, 43]. If conservation biologists are to achieve rates of ecopassage usage as high as those reported for arena studies, the associated exclusion structure needs to remove any other crossing option (e.g., crossing over, under, through gaps in the fence, or circumnavigation around the fence). To achieve this goal, exclusion structures guiding individuals toward ecopassages should remain continuous beyond the target species’ dispersal distance [37], rather than merely the length of suitable habitat (e.g., the distance along which suitable habitat abuts a road). Without other crossing options, wildlife should be far more likely to use an ecopassage. In our study, as a result of the numerous failures in the exclusion structure at the Impact site, turtles that refused to use the ecopassage were provided with a multitude of other crossing options. We urge future studies to examine how other reptile species, particularly squamates, and small to medium-sized mammals, respond to ecopassages as these areas of research are sorely lacking.

Due to the high cost associated with the installation of ecopassages, it is important that their likelihood of use is ensured. Through better understanding of an animal’s willingness to use ecopassages and species-specific dispersal distances between and around ecopassages, conservation biologists and wildlife managers can more effectively design mitigation measures that will work properly, in turn optimizing the structure’s ecological value and offsetting its initial monetary costs.

### Prevention of prey-trap formation (criterion 3)

A common concern regarding crossing structures is their potential to be prey-traps [32]. We observed no predation of reptiles within the ecopassages. Yet, of the observed wildlife in the ecopassages (*n* = 485 observations), 22.3% were known reptile predators [25, 49]. In contrast, reptiles only accounted for 2.8% of observed wildlife within the ecopassages. Thus, at the current level of reptile use, the ecopassages in our study would be highly ineffective for a predator to use as a hunting location. Most likely, predator presence in the ecopassage is simply related to road crossings. Similar findings refuting the prey-trap hypothesis have been noted for both small and large mammals [32, 53]. However, if reptile abundance was to be increased within the ecopassages, then further examination of the potential for prey-trap formation would be required.

## Conclusion

The use of road mortality mitigation measures is crucial for the conservation of biodiversity [1, 7, 8]. However, the specific mitigation measures we tested were deemed ineffective because of their inability to reduce the abundance of reptiles on the road, and the limited use of the ecopassages. Our study further demonstrates that conservation decisions need to be supported by solid science, and it is critical that highway designers and wildlife managers rigorously and thoroughly test the effectiveness of mitigation measures [7, 29]. Our study is important because it examined the effectiveness of the mitigation measures currently being implemented into road projects, and did so with a taxa-wide approach. Unlike previous studies that focused on key species and specific aspects of demographic differences in crossing rates and locations, our study takes the approach that if mitigation measures are to be deemed effective, they should be able to reduce overall percentage of dead reptiles on the road, maintain connectivity, and prevent prey-trap formation regardless of changes in road width, speed limits, and inter-population variability in crossing behavior. If a road is to be considered mitigated for a group, or better yet, multiple groups of species, then the mitigation measures used should result in an overall functional level of protection for the complement of populations, including all demographic subgroups.

As global biodiversity decreases [54] and threats to animal populations are identified and become more wide-spread, we must strive to increase our level of protection for rare and imperiled species beyond current norms. Furthermore, mitigation methods should also be designed to offer protection to common species. Mitigation is more than a political mandate satisfying a piece of legislation, it is a means to create infrastructure that directly reduces the negative impacts of development on wildlife [17]. Mitigation measures should be designed to last over the long-term (i.e., the lifespan of the road). Materials used for exclusion structures need to be enduring (e.g., concrete gravity walls, solid steel barriers), and should be incorporated into highway engineering and provided the same level of scrutiny given to road construction. The effectiveness of population connectivity structures relies on the effectiveness of exclusion structures. Although we observed some use of ecopassages, it occurred at low rates, particularly because other, albeit more risky, crossing options existed. Ecopassages are effective and worth their monetary cost when the associated exclusion structures are working. If care is taken to properly assess the effectiveness of mitigation, and steps are taken to adaptively manage the negative impacts of roads, we can further our efforts to stem the tide of species loss, and better conserve and protect the natural world.

## Supporting Information

S1 Table Animals observed crossing through ecopassages under the highway. Vertebrate fauna passing through three ecopassages under Highway 69, Burwash, Ontario, Canada, were recorded on wildlife cameras from 1 May to 31 August 2013. Crossings are indicated by number of photos taken and percentage of total photos taken.
(DOCX)
