# Supplementary material for: Mitigating Reptile Road Mortality: Fence Failures Compromise Ecopassage Effectiveness
Source: PLoS One. 2015 Mar 25;10(3):e0120537. doi: 10.1371/journal.pone.0120537 (PMC4373904; doi:10.1371/journal.pone.0120537)
Supplement: S1 Table — Vertebrate fauna passing through three ecopassages under Highway 69, Burwash, Ontario, Canada, were recorded on wildlife cameras from 1 May to 31 August 2013. Crossings are indicated by number of photos taken and percentage of total photos taken. (DOCX) [file pone.0120537.s001.docx]

**S1 Table. Animals observed crossing through ecopassages under the highway.** Vertebrate fauna passing through three ecopassages under Highway 69, Burwash, Ontario, Canada, were recorded on wildlife cameras from 1 May to 31 August 2013. Crossings are indicated by number of photos taken and percentage of total photos taken.

| **Species or Group** | **Number of photos** | **Percentage of total photo captures (%)** |
| --- | --- | --- |
| Dabbling Ducks (*Anas* sp.) | 115 | 23.7 |
| Canada Goose (*Branta canadensis)* | 80 | 16.5 |
| Hare/Rabbit (Order: Lagomorpha) | 51 | 10.5 |
| Great Blue Heron (*Ardea herodias)* | 43 | 8.9 |
| Raccoon (*Procyon lotor)* | 40 | 8.3 |
| Muskrat (*Ondatra zibethicus)* | 30 | 6.19 |
| White-tailed Deer (*Odocoileus virginianus)* | 25 | 5.2 |
| North American Beaver (*Castor canadensis*) | 17 | 3.5 |
| Passerine Birds (Order: Passeriformes) | 15 | 3.1 |
| Frogs (*Lithobates* sp.) | 14 | 2.9 |
| Mink (*Neovison vison*) | 11 | 2.3 |
| Coyote/Wolf (*Canis* sp.) | 8 | 1.7 |
| Red Fox (*Vulpes vulpes*) | 7 | 1.4 |
| Painted Turtle (*Chyrsemys picta*) | 6 | 1.2 |
| North American River Otter (*Lontra canadensis*) | 5 | 1 |
| Black Bear (*Ursus americanus*) | 4 | 0.8 |
| Porcupine (*Erethizon dorsatum)* | 4 | 0.83 |
| Northern Watersnake (*Nerodia sipedon*) | 3 | 0.6 |
| Groundhog (*Marmota monax)* | 2 | 0.4 |
| Small mammals (Orders: Rodentia and Soricomorpha) | 2 | 0.4 |
| American Red Squirrel (*Tamiasciurus hudsonicus)* | 1 | 0.2 |
| Moose (*Alces alces*) | 1 | 0.2 |
| Snapping Turtle (*Chelydra serpentina*) | 1 | 0.2 |
| **Total** | **485** |  |
